# Comparative Evaluation of Treatment Outcomes of Revision Anterior Cruciate Ligament Reconstruction Using Allograft and Semitendinosus Autograft

**DOI:** 10.3390/jcm14010133

**Published:** 2024-12-29

**Authors:** Maciej Kentel, Monika Kentel, Krzysztof Korolczuk, Jarosław Witkowski

**Affiliations:** 1 eMKaMED Medical Center, 53-110 Wroclaw, Poland; maciej.kentel@emkamed.com.pl (M.K.); monika.kentel@emkamed.com.pl (M.K.); 2 Department of Orthopedics, Traumatology and Hand Surgery, Faculty of Medicine, Wroclaw Medical University, 50-556 Wroclaw, Poland; krzysztof.koroloczuk@umw.edu.pl

**Keywords:** ACLR—ACL reconstruction, RACLR—revision ACL reconstruction, ST—semitendinosus, graft, arthroscopy

## Abstract

**Introduction:** The number of revision anterior cruciate ligament reconstruction (RACLR) procedures is increasing in proportion to the increase in the number of anterior cruciate ligament reconstruction (ACLR) procedures. Although approximately 50–75% of these procedures can be performed in a single-stage procedure, not all of them can. The choice of graft may influence the results of RACLR. The most commonly mentioned graft materials for RACLR are allografts and autografts. **Background/Objectives**: The aim of the study was to evaluate the results of single-stage RACLR using a semitendinosus (ST) autograft or allograft and to follow-up and compare the results of both groups after 2 years. **Methods**: The retrospective cohort study was carried out between 2008 and 2021, during which time 2327 ACLRs were performed. Graft rupture occurred in 198 (8.5%) patients, and 98 (4.2%) patients underwent RACLR. RACLR was performed as a single-stage procedure using a semitendinosus autograft or allograft in 56 patients. The KT-2000, Lachman and axis shift, range of motion, Tegner, Lysholm, KSS, KOOS, and IKDC tests were used to assess outcomes. **Results**: There were no differences between the groups in terms of age, time of revision after procedure, parameters for the graft (screw diameter, endobutton length, femoral tunnel length) or for the procedure and revision, BMI, or in the time needed for returning to dynamics and training. The groups did not differ in quality of life in any measurements, stiffness, pain function, or sport. Analysis showed higher Lysholm results on the day of treatment in the autograft group than in the allograft group (*p* = 0.11). The allograft group had higher KSS scores on the day of treatment (*p* = 0.11) and after 60 months or at the moment of breakup than the autograft group (*p* = 0.025). **Conclusions**: Single-stage revision anterior cruciate ligament reconstruction with an autograft and ST provides good knee stability. The results of single-stage revision anterior cruciate ligament treatment using an ST autograft or an ST allograft are similar.

## 1. Introduction

Anterior cruciate ligament (ACL) rupture is a common knee joint injury that requires surgical treatment [1,2]. There are approximately 200,000 ACL failures per year in the USA [3]. Anterior cruciate ligament reconstruction (ACLR) is a burden, with a constant number of failures and complications and with 2.5–27% of end grafts that rerupture [2,4]. Primary ACL reconstruction failures are multifactorial [1], with reasons stemming from patient demographics (age, gender, body mass index), sport practiced, level of competition, and the presence of omitted and/or concomitant injuries of cartilage, cruciate and collateral ligaments (and the meniscus), technical issues (type, size, and tension as well as transplant and tunnel preparation) and postoperative management (rehabilitation compliance and complications) [1]. Revision anterior cruciate ligament reconstruction could improve clinical and stability results; however, the clinical results were inferior to those of primary reconstruction [5]. Patients undergoing revision ACLR with an autograft can be expected to experience lower rates of graft retear, higher rates of return to sports, and less postoperative anteroposterior knee laxity when compared with patients undergoing revision ACLR with an allograft [6].

ACLR is a consequence of repeated trauma (10–35%), technical error (20–40%), biological failures (5–15%), or a combination of factors (32–45%) [4,7]. After repeated ACL graft damage, a proportion of patients, from 5–30%, accept dysfunction of the knee joint [8,9]. Physically active patients choose reoperation—revision ACL reconstruction (RACLR) [7,9,10]. RACLR results are inferior to original ACL reconstructions, with 57% of patients returning to sports from before the injury [9,10]. The number of RACLR procedures increases proportionally with the increase in ACLR procedures [7,11]. Although approximately 50–75% of the procedures can be performed in one-stage mode, not all of them can [7,8,9,10]. Graft selection may affect the RACLR results. The most frequently mentioned materials for RACLR grafts are allografts and autographs [1,4,11].

After having anterior cruciate ligament (ACL) reconstruction, which often involves using hamstring tendons like the semitendinosus and gracilis tendons, extensive postoperative physiotherapy is necessary to restore knee function. Research indicates that longer periods of supervised rehabilitation lead to better outcomes in agility, strength, and symmetry during activities such as vertical jump landings [12,13,14,15,16,17,18,19]. Furthermore, early kinematic assessments reveal significant muscle adaptations and emphasize the importance of addressing between-limb asymmetry for long-term recovery [20].

## 2. Objectives

The aim of the study was to evaluate the results of single-stage revision anterior cruciate ligament reconstruction (RACLR) using a semitendinosus (ST) autograft or allograft and to follow-up and compare the results of both groups after 2 years.

## 3. Materials and Methods

### 3.1. Materials

**Figure 1:**
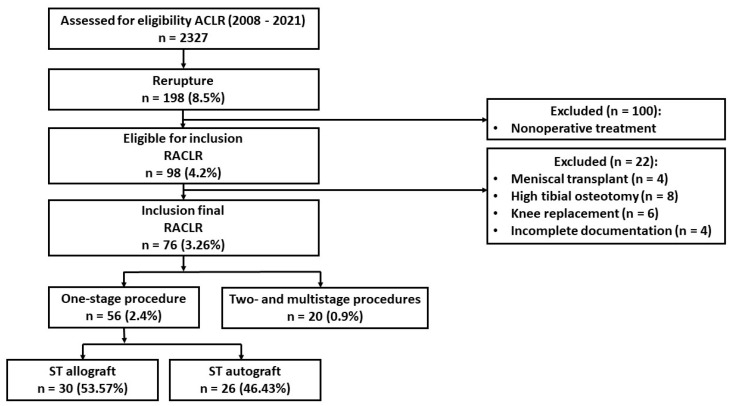
Graphical evaluation of the study group.

From 2008 to 2021, 2327 ACLRs were performed. In 198 (8.5%) patients, the graft ruptured again, and RACLRs were performed in 98 (4.2%) patients. For this analysis, we eliminated patients who underwent nonoperative treatment (n = 101), reoperations that included meniscal transplant (n = 4), high tibial osteotomy (n = 8), and knee replacement (n = 6). While four patients had incomplete documentation, full documentation was obtained for 76 (3.26%) patients. In 56 (73.68%) patients, RACLR was performed in a one-stage procedure, and two- and multi-stage procedures were performed in 20 patients (26.32%). Figure 1 shows a graphical evaluation of the study group.

After qualifying the patients for surgical treatment, based on the above criteria, and getting their consent to perform this method of treatment, the choice of graft was discussed with them. A double-folded ST allograft or autograft was proposed as an alternate. The final decision regarding the graft was made by the patients after the operating physician had presented the available options and their advantages and disadvantages. In autograft revision surgery, the semitendinosus was used from the contralateral knee. Until 2017, the Smith & Nephew (S&N) bioabsorbable screw was used to stabilize the material; however, from 2018 onward, FastThread Arthrex bioabsorbable screws were used. We compared the sick joint with the healthy joint using the KT-2000, Lachman test, and pivot shift test, and we assessed the compactness of the joint after the procedure on the day of surgery and after 12 and 60 months. We measured the range of motion, assessed the patients’ activity using the Tegner scale, and assessed clinical results using the Lysholm, KSS, KOOS, and IKDC scales. Validation was performed by native-speaking researchers, based on scales that were translated into Polish and that were the same for all respondents. The criteria that made it possible to perform single-stage RACLRs are shown in Table 1 [21].

In the group of 56 (2.4%), RACLR grafts were used (mean age 30.2 years):

- Allograft in 30 (53.57%); 25 men; mean age 30.43
- Autograft in 26 (46.43%); 21 men; mean age 30.15

Detailed analyses of any failures were performed in all of the instances, taking into account the causes (Table 2 and Table 3):

- Graft—type, diameter, material
- Surgical technique
- Type of attachment
- Collateral ligaments
- Other coexisting injuries
- Sports
- Age
- BMI

This study was carried out according to the principles of the Helsinki Declaration and approved by the Bioethics Committee of Wroclaw Medical University. All of the participants were informed about the study’s aim and applied approach, and all signed their informed consent forms for participation. An additional goal of the assessment was to present the patients with the technique to be applied.

### 3.2. Description of the Procedure

After the patients qualified for the procedure, a detailed plan was developed to anticipate possible difficulties or complications. Joint compactness was assessed in all planes (KT-1000), as was range of motion and axis of the knee joint. The procedure was performed under spinal anesthesia, with the patient positioned on his or her back, with supports under the thigh to allow the joint to bend the knee up to about 120°. Ischemia was provided by an Esmarch band of 250–270 mm Hg. The average treatment time was approximately 70 min.

**Figure 2:**
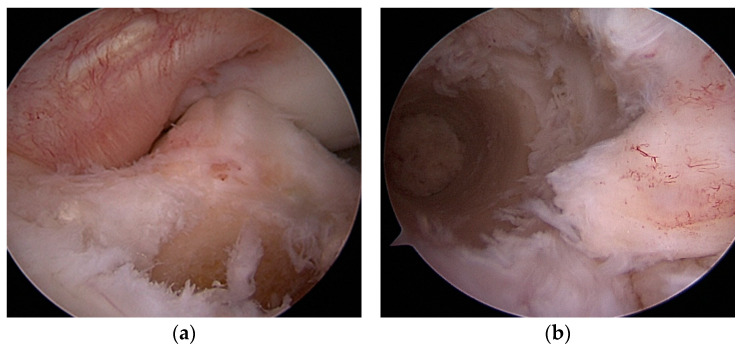
(**a**,**b**) Preparing new tibial and femoral canals.

**Figure 3:**
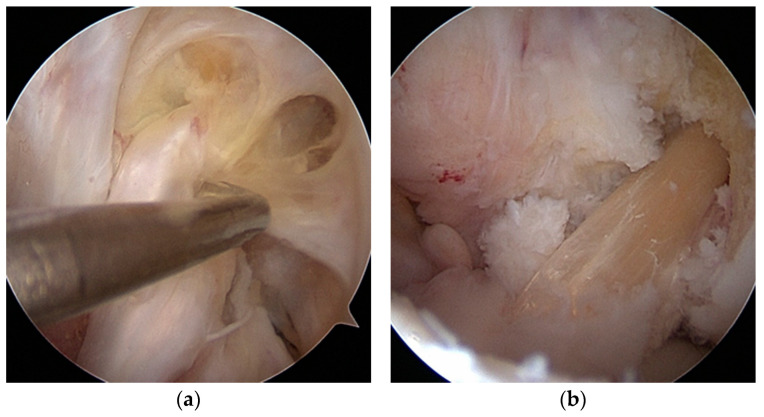
(**a**) Wrong positioning of femoral attachment in primary ACLR. (**b**) Correct femoral attachment.

In the first stage, after the arthroscope was inserted into the joint, remnants of the ACL graft were removed. Any meniscal and cartilage lesions were debrided, and the femoral and tibial tunnels were assessed for correct placement. The tibial tunnel was prepared by removing the remnant screw. The anatomical tibial insertion site of the ACL was then identified using a targeting device. A Kirschner wire was inserted into the tunnel from the outside over the targeting device and drilled to the tibial insertion site inside the joint. The tunnel was reamed, and the drill diameter was gradually increased by removing the remnants of the bioabsorbable screw and graft. The tunnel was reamed until healthy cancellous bone was obtained. In all patients operated on using the single-stage method, the tibial tunnel was correctly positioned, and its diameter did not exceed 10 mm. The femoral insertion was then identified. If the original femoral tunnel was incorrectly positioned, a new one was drilled; a guide wire was inserted from the outside into the joint to place the correct insertion using a transtibial technique (Figure 2 and Figure 3). If the femoral attachment was anatomically placed, the knee joint was flexed 100–110 degrees, and a new canal was drilled outward. In this way, the canal was made at a different angle than the previous one. Drilling was also performed using the transtibial technique. The canal was then reamed in the usual manner adapted to the graft diameter.

**Figure 4:**
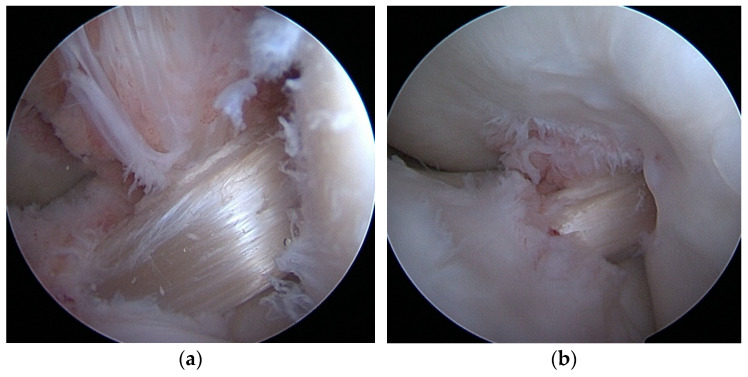
(**a**,**b**) Revision ST autograft.

**Figure 5:**
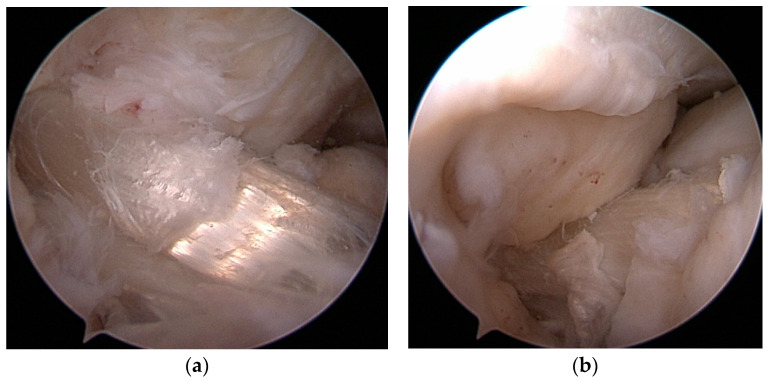
(**a**,**b**) Revision ST allograft.

A graft was prepared using the semitendinosus muscle tendon. In autograph cases, the tendon was taken in a typical manner from the same knee—or from the other knee—if the tendon had already been used in the original reconstruction. In allograft cases, the tendon was freshly frozen and obtained from a tissue bank (and tested for infectious diseases and HIV). In both cases, the tendon was placed on the preparation table twice—once for a diameter adjustment and then for a length adjustment—and sewn in a typical way. The tendon was adjusted from 8 to 9.5 mm in diameter and then from 65 to 85 mm in length, depending on the size needs. The graft was introduced into the joint from the tibial canal, stabilized on the thigh with an endobutton hanger, and then stretched by bending and straightening the joint, checking the isometry (Figure 4 and Figure 5).

The endobutton loop length was adjusted to a length 23 mm shorter than the tibial canal. The tibia trailers were stabilized using an S&N bioabsorbable screw. After treatment, two drains were left in the joint cavity and tibial trailer for one day.

Following the RACLR, all patients received the same treatment rehabilitation protocol, considering the treatment of comorbidities and injuries.

All patients were treated with a dynamic knee brace immediately after surgery for 6 weeks, with a 6-week range of motion restriction (weeks 1–2: 30° knee flexion, weeks 3–4: 60° knee flexion, weeks 5–6: 90° knee flexion). Physiotherapy began on the first day after surgery, and full extension, quadriceps stimulation, and anti-edema therapy were initiated as soon as possible. After the sixth week, measurements were performed on the Gamma dynamographic platform using the Up & Go test, range of motion measurements, and muscle circumferences. In the cases where patients regained a full range of motion, joint compactness, a difference in muscle circumferences of no greater than 1.5 cm, and symmetrical loading of the limbs in a free standing position, these patients were allowed to proceed to the next stage of physiotherapy consisting of rebuilding the muscle corset, working on restoring proprioception, and introducing preparation for returning to sports.

The return of strength and muscle endurance of the operated limb, as confirmed by positive results of strength measurements in static conditions (weeks 12–14) and by measurements on the Gamma dynamographic platform (free standing test, step test, standing and sitting test), allowed the patient to move to the next stage of physiotherapy—preparing for a return to sports. Patients returned to sports without rotation (running, cycling, etc.) after six months.

First, dynamic loads and elements of a given discipline were introduced. In weeks 16–18, the first measurements of moments of force were performed in isokinetic conditions on Biodex. In the case of differences in strength, power, and total work of less than 10%, the next stage was allowed, i.e., returning to sports at the initial level—before the ligament injury—after nine to twelve months of healing time.

Approved clinical measurements, such as the IKDC result, KT-1000 measurements, Lachman degrees, and degrees of axis change were taken. Data collected included baseline demographics, surgical techniques and pathology, and a series of validated patient-reported outcome measures (IKDC, KSS, Lysholm, Tegner, and KOOS). Patients were followed for two years and asked to complete an identical set of outcome measurements.

### 3.3. Data Analysis

Data analyses were carried out using IBM SPSS Statistics 27.0. The significance level was α = 0.05. Generalized linear models (GLMs) with Gamma distribution probability and a log linking function were performed for the allograft group and with linear distribution probability and an identity linking function for the autograft group. The elapsed time between surgery and revision was included as the model’s dependent variable, while diameter, screw, endobutton, age at surgery, and BMI differences between surgery and revision were included as predictor variables. The model’s fit to the data was determined using the Omnibus test, which determines whether the model better predicts the dependent variable than the intercept.

To compare the autograft and allograft groups in terms of perioperative parameters, quality of life, and exercise performance, the Mann–Whitney U test was performed.

## 4. Results

### 4.1. Autografts

To determine the durability of the procedure performed in autograft patients, generalized linear models for the probability of linear line conversion with the identity linking function were applied. The omnibus test was ultimately irrelevant (x^2^(5) = 17.16; *p* = 0.209), which indicates that the fitted model did not differ significantly from the intercept-only model. Therefore, such a model is not subject to interpretation and requires modification. Neither the model containing only the graft data (diameter, screw, and endobutton; x^2^(3) = 5.96; *p* = 0.114) nor the model containing metric parameters (∆BMI and age; x^2^(2) = 1.95; *p* = 0.377) fit the data.

### 4.2. Allografts

The omnibus test was statistically significant (x^2^(5) = 18.36; *p* = 0.003), indicating an adequate fit of the model to the data (AIC = 294.99; deviance = 0.47). The analysis showed significant effects on the endobutton (*p* = 0.001), ∆BMI (*p* = 0.006), and age at the time of surgery (*p* = 0.002). The higher the endobutton value, the older the subjects, the smaller the difference between the BMI measurements, and the longer the time from surgery to revision. Detailed parameter estimates are presented in Table 4.

### 4.3. Autograft and Allograft Comparisons

There were no differences between the groups in terms of age, time of revision after procedure, parameters for the graft or for the procedure and revision, BMI, or in time needed for returning to dynamics and training. Table 5 presents the comparison in terms of periprocedural parameters. Analyses were performed using the Mann–Whitey U test.

The groups did not differ in quality of life in any measurements. Table 6 presents the comparison of the KOOS scale results, which includes parameters such as quality of life, stiffness, pain function, and sport.

Analysis showed higher Lysholm results on the day of treatment in the autograft group than in the allograft group (p = 0.11). The allograft group had higher KSS scores on the day of treatment (*p* = 0.11) and after 60 months or at the moment of breakup than the autograft group (*p* = 0.025). The differences between the groups were moderate. Table 7 presents the comparative analyses in terms of exercise performance.

## 5. Discussion

Age is a significant factor in ACLR outcomes. The mean patient age was 30.15 +/− in the autograft group and 30.43 +/− in the allograft group. In our group of patients, we did not notice any breakup grafts. Our results align with the statistics of reports showing less susceptibility to graft failure with increasing age. This is possibly because of less physical activity in this age group. Wasserstein et al. [22] recorded repeated autograft damage at 9.6% and allograft damage at 25% in the third follow-up in the group of 21.7-year-olds, which was the youngest group. In the MARS group, the median age was 26 years. The authors indicate that failure is an age-dependent issue. As age increases, the risk of damage decreases; a 35-year-old has a 59% lower risk of breaking a graft than a 20-year-old [23]. Other studies in cohorts with primary ACLRs have shown that younger age and higher baseline activity levels are predictors of an increased failure rate [24,25,26,27,28]. Samuelsen et al. [29] evaluated a cohort of 7560 patients (mean age 28.5 years) and noted that a lower age was associated with an increased risk of recurrent ligament disruption on both the ACLR and contralateral sides. A similar age group was analyzed by Grassi et al. [30], who analyzed patient groups from 30.4 years of age, concluding with a negligible failure rate.

### 5.1. Graft Size

Graft size was ultimately not a variable predictor. We found no correlation between the graft diameter and results. The mean size of the autografts and allografts was 9 mm in the tibial canal and 8 mm in the femoral canal. Sasaki et al. [31] quoted the following initial dimensions: 6.2 mm of AM and 5.9 mm of PL in two-bundle reconstruction and 10 mm in the BPTB graft (with a need to widen the graft and canals by 1–2 mm). The reports agree that in the case of revision reconstruction, a larger diameter graft is necessary [8,10,11,24,32,33,34]. Our analysis confirms these reports—we noted 1 mm increases in the revision prosthesis diameter and canal width. This applied to both groups.

### 5.2. Tibia Stabilizing Screw Dimensions

The tibia stabilization screw dimensions for autograft stabilization were 1 mm larger during the revision reconstruction (7–9 mm before and 8–10 mm after). The same dimensions were obtained in the allograft group, and this did not affect the clinical outcomes and recovery. Numerous authors have emphasized the possibility of using spongy bone transplants in the case of existing defects in the tibial canal, but this was unnecessary with our material.

For femur stabilization, the loop size was shortened by an average of 10 mm (the endobutton was 35 mm in first surgery and 25 mm at revision reconstruction). This relationship was the same in groups I and II.

In the allograft group, we observed a relationship between endobutton pendant length during the primary reconstruction and revision time. The longer the original endobutton, the longer the time to secondary graft failure and revision reconstruction. The mean survival of the graft was approximately 110 months for a 45 mm loop length and approximately 70 months for a 40 mm loop length. The mean time from primary reconstruction in this group was 57.44 weeks. These observations were not confirmed in group I. The longer endobutton loop dimensions were forced by longer femoral canals. The inserted ligament graft length was the same as the shorter loops and ranged from 15 to 20 mm. In future research, we will analyze the possibility that the greater flexibility of the longer loop contributed to better healing and graft reconstruction.

### 5.3. BMI

We recorded a BMI increase from 24.9 on the day of reconstruction to 26.37 on the day of revision in the group I autografts. In the allograft group, this ranged from 25.25 on the day of reconstruction to 26.54 on the day of revision. In group II, we observed a correlation—the smaller the difference between the BMI measurements, the longer the time from treatment to revision. The BMI increase between the time of primary reconstruction and return to physical fitness has been mentioned by many authors as a factor of graft disruption [8,24,27,30,32,33,35,36,37]. This was confirmed in our observations. This issue requires emphasis and further analysis.

### 5.4. Time from Basic ACLR to Revision

In our study, the time from primary reconstruction to revision was 45.1 months. We observed no correlation between time and the primary results of time reconstruction and reconstruction in group I. Wright et al. [2] made similar observations with a median time of 39 months.

In group II, the analysis showed a significant influence on the relationship between the endobutton loop length, weight gain, and age at the time of surgery. The longer the endobutton loop, the older the subjects. Moreover, the smaller the difference between the BMI measurements, the longer the time from surgery to revision.

A longer endobutton hanger loop in the primary group will be the subject of further observation and analysis. The younger the age, the higher the physical activity level. There are ongoing observations regarding the necessity of supplying secondary stabilizers to the knee joint. An increased BMI is also an indicator of graft breakage risk. The inability to return to activity, make lifestyle changes, and change eating habits given the availability of food are all factors that influence BMI increase. In our opinion, education recommending changing all groups’ eating habits and limiting activity until healed in the group of younger athletes decreases the number of failures.

We found that autologous transplants resulted in better Lysholm scores on the day of surgery (*p* = 0.011) but that allografts resulted in better KSS scores on the day of surgery (*p* = 0.001) and at 60 months (*p* = 0.025). Beyond these differences, our study indicates that there is no significant difference between autografts and allografts in periprocedural parameters. In neither group was the graft damaged. Therefore, based on these data, we cannot say which type of transplant is better. However, a 2021 study found that the use of a bone–patellar tendon–bone autograft (BPTB) for revision resulted in a 4.2-fold lower probability of maintaining a subsequent graft fracture in patients than with the BPTB allograft (*p* = 0.011). This study analyzed BPTB and soft tissue autografts, as well as BPTB and soft tissue allografts. Both the BPTB and the soft tissue autografts failed at a much lower frequency than did the BPTB allografts (*p* = 0.016 for both) [38]. A 2017 study found that autograft use results showed lower postoperative flaccidity, complication rates, and repeat surgeries. After excluding patients who received irradiated allografts, the results for autografts and allografts were similar. Non-irradiated transplants had better results than BPTB autografts in terms of complications and reoperations [30]. Data from 2019 show that patients who received allografts for ACL revisions were 2.78 times more likely to break a graft than those who received autografts. These data also confirm that non-irradiated allografts provide similar results to autologous transplants [39]. In 2015, 1016 patients were examined after ACLR. The overall failure rate was 9.6% (76/788) for autologous transplants and 25.0% (57/228) for allografts [22], which supports the success of autologous transplants.

We found no difference in knee joint stability between the groups. The Lachman and KT-1000 tests, page to page, were comparable.

The answer as to which type of transplant predisposes a better return to sport is ambiguous. We found that allografts resulted in better KSS scores on the day of surgery (*p* = 0.001) and at 60 months or at the time of rupture (*p* = 0.025) and that autologous transplants resulted in better Lysholm scores on the day of surgery (*p* = 0.001). In the IKDC, KOOS, and TEGNER subscales, graft selection was an irrelevant factor in predicting results immediately after surgery and 12 and 60 months after surgery.

Another study involving 809 patients found that transplant type was a significant predictor of Marx’s six-year activity level (*p* = 0.024). The authors of the study also state that in the IKDC, KOOS, and WOMAC subscales, transplant choice was an insignificant factor in predicting six-year treatment outcomes [38]. Earlier studies of 989 patients showed that graft selection is a significant predictor of the two-year IKDC results (*p* = 0.017). Autograft use for revision reconstruction predicted IKDC score improvement (*p* = 0.045) and KOOS quality of life subscale result improvement two years after the procedure (*p* = 0.031) [23]. Higher Lysholm scores for autografts have also been reported in the literature (*p* = 0.045) [40,41].

To date, studies evaluating the results of the five-year ACL revision reconstruction are limited. According to the current literature, autografts have a lower risk of graft rupture compared with allografts. Autografts are associated with higher levels of activity and less postoperative laxity than allografts [22,23,30,38,39]. Experts say that the choice of graft for ACLR depends primarily on the surgeon’s preferences. The choice of graft for RACLR surgery depends on the previously used graft and tunnel dimensions, as these considerations indicate greater effectiveness of autografts [23,40].

### 5.5. Limitations

The main limitation of this study was no randomization. After being presented with both ACLR procedures, the patients could choose the one they wanted. The limitations include the retrospective design, potential biases from patient graft choice, and the exclusion of multi-stage RACLR cases. We see a need for prospective research.

## 6. Conclusions

Single-stage RACLR with a semitendinosus autograft or allograft (ST) provides good knee stability. The allograft and autograft procedures did not differ in terms of age, revision time after the first procedure, graft parameters (screw diameter, endobutton length, femoral tunnel length), BMI, or a return to sports. The groups did not differ in terms of quality of life, stiffness, pain, and sports function. Differences were observed in Lysholm and KSS scores.

## Figures and Tables

**Table 1:** Type of graft in the study group in primary reconstruction.

Single-Stage ACL Revision	Two-Stage ACL Revision Surgery
Good range of motion	Stiff knee (>20° flexion deformity, <80° flexion)
Tunnel dilation < 14 mm	Tunnel dilation > 14 mm
Completely incorrect or completely correct tunnel	Partially correct tunnel (2/3 overlap with ideal position)
Smaller coronal plane deformity requiring correction (<10°)	Large coronal plane deformity requiring correction (>10°)
Posterior tibial slope ≤ 10°	Posterior tibial slope > 12
Operating time < 90 min	Operating time > 90 min

**Table 2:** Type of graft in the study group in primary reconstruction.

Type of Graft	Quantity (56)
Autograft 2 × HT (hamstring)	48
Ligament repair and reconstruction solutions (LARS)	4
JewelACL	2
Allograft HT × 2	2

**Table 3:** The reasons for graft failure in primary reconstruction.

The Reasons for the Failure—Intraoperative Analysis	Quantity (56)
Injury	40
Malposition of femoral tunnel	5
Incorrect tension of ACL graft	3
Insufficiency all (rotator instability)	3
Destruction of LARS	4
Tibial fatigue fracture	1

**Table 4:** Parameter estimates for the model explaining the time from surgery to revision.

95% CI							
Parameter	B	SE	LL	UL	X^2^Walda	df	*p*
Intercept	1.25	2.56	−3.83	6.33	0.23	1	0.630
Diameter	−0.24	0.25	−0.72	0.24	0.95	1	0.329
Screw	0.05	0.27	−0.49	0.58	0.03	1	0.859
Endobutton	0.08	0.02	0.04	0.13	11.30	1	**0.001**
∆BMI	−0.35	0.13	−0.60	−0.10	7.42	1	**0.006**
Age at the time of surgery	0.05	0.02	0.02	0.08	9.89	1	**0.002**
Scale	0.36	0.09	0.22	0.58			

B—custom regression coefficient. SE—standard error. LL and UL—lower and upper limits of the confidence interval. x^2^Walda—Wald test statistic. df—degrees of freedom. *p*—test probability. 95% Cl—95% confidence interval.

**Table 5:** Group comparisons in terms of periprocedural parameters.

	Allograft (n = 30)			Autograft (n = 26)			Z	*p*	r
	Average Rank	Me	IQR	Average Rank	Me	IQR			
Age at the time of surgery	28.77	28	14	28.19	28.50	9.75	−0.13	0.895	0.02
Time from surgery to revision	32.10	37	56.75	24.35	31.50	31.75	−1.77	0.076	0.24
**Surgery**									
Screw	31.13	8.00	0.50	25.46	8.00	0.50	−1.41	0.158	0.19
Diameter	29.28	9.00	0.25	27.60	9.00	1.00	−0.50	0.618	0.07
Endobutton	29.48	35.00	5.00	27.37	35.00	5.00	−0.51	0.612	0.07
**Revision**									
Screw	28.47	9.00	0.50	28.54	9.00	0.50	−0.02	0.985	0.00
Diameter	29.38	10.00	1.00	27.48	10.00	1.00	−0.53	0.597	0.07
Endobutton	28.50	27.50	5.00	28.50	27.50	5.00	0.00	1.000	0.00
BMI surgery	29.53	25.58	3.01	27.31	25.59	3.87	−0.51	0.610	0.07
BMI revision	28.43	27.00	3.32	28.58	27.30	3.34	−0.03	0.974	0.00
∆BMI	28.82	1.10	0.56	28.13	0.99	0.79	−0.16	0.876	0.02
Time to return to dynamics [months]	27.88	5.00	2.00	29.21	5.50	2.25	−0.31	0.753	0.04
Time to return to training [months]	32.30	11.00	3.00	24.12	9.00	2.00	−1.91	0.056	0.26

Me—mean. IQR—interquartile range. Z—standardized Mann–Whitney U statistics. *p*—test probability. r—effect size.

**Table 6:** KOOS scale comparison.

	Allograft (n = 30)			Autograft (n = 26)			Z	*p*	r
	Average Rank	Me	IQR	Average Rank	Me	IQR			
**Measurement I**									
Stiffness	28.20	39.29	10.71	28.85	39.29	3.15	−0.16	0.869	0.02
Pain	27.10	55.56	0.00	30.12	55.56	0.00	−0.93	0.351	0.12
Function	29.30	52.94	22.06	27.58	52.94	22.06	−0,44	0.659	0.06
Sport	29.20	5.00	15.00	27.69	5.00	5.00	−0.41	0.684	0.05
Quality of Life	26.03	43.75	6.25	31.35	43.75	0.00	−1.46	0.144	0.20
**Measurement II**									
Stiffness	29.28	71.53	26.49	27.60	71.43	25.79	−0.42	0.678	0.06
Pain	28.00	91.67	5.56	29.08	91.67	5.56	−0.28	0.776	0.04
Function	28.53	94.12	3.48	28.46	95.59	3.48	−0.02	0.986	0.00
Sport	29.68	75.00	10.00	27.13	75.00	15.00	−0.60	0.549	0.11
Quality of Life	28.35	93.75	12.50	28.67	93.75	12.50	−0.09	0.931	0.02
**Measurement III**									
Stiffness	29.57	71.43	25.79	27.27	71.43	25.79	−0.57	0.568	0.10
Pain	28.90	86.11	5.56	28.04	88.89	5.56	−0.21	0.835	0.04
Function	30.20	94.12	3.48	26.54	94.12	3.10	−0.90	0.367	0.16
Sport	31.37	70.00	15.00	25.19	70.00	12.50	−1.44	0.149	0.26
Quality of Life	28.80	81.25	12.50	28.15	81.25	12.50	−0.16	0.872	0.02

Me—mean. IQR—interquartile range. Z—standardized Mann–Whitney U statistics. *p*—test probability. r—effect size.

**Table 7:** Comparison in terms of exercise performance (Lysholm, TEGNER, KSS, and IKDC).

	Allograft (n = 30)			Autograft (n = 26)			Z	*p*	r
	Average Rank	Me	IQR	Average Rank	Me	IQR			
Lysholm on the day of surgery	23.40	35.50	7.00	34.38	40.00	7.00	−2.55	**0.011**	0.34
Lysholm after 12 months	27.38	96.00	8.00	29.79	97.00	8.00	−0.56	0.575	0.07
Lysholm after 60 months or at breakup	30.03	95.00	7.50	26.73	92.50	12.75	−0.76	0.446	0.10
Tegner on the day of surgery	28.62	7.00	2.00	28.37	6.50	2.00	−0.06	0.953	0.01
Tegner after 12 months	25.77	6.00	2.25	31.65	6.00	3.00	−1.37	0.171	0.18
Tegner after 60 months or at breakup	26.63	6.00	1.50	30.65	6.00	3.00	−0.94	0.347	0.13
IKDC on the day of surgery	29.10	44.80	8.00	27.81	44.80	8.00	−0.34	0.736	0.05
IKDC after 12 months	28.43	85.10	12.60	28.58	91.25	12.60	−0.03	0.973	0.00
IKDC after 60 months or at breakup	30.83	85.10	12.60	25.81	85.10	12.60	−1.27	0.204	0.17
KSS on the day of surgery	35.13	64.00	10.00	20.85	62.00	2.75	−3.38	**0.001**	0.45
KSS after 12 months	30.82	93.00	2.00	25.83	92.00	3.00	−1.18	0.237	0.16
KSS after 60 months or at breakup	32.90	94.00	2.00	23.42	91.50	3.25	−2.24	**0.025**	0.30

Me—mean. IQR—interquartile range. Z—standardized Mann–Whitney U statistics. *p*—test probability. r—effect size.

## Data Availability

No new data were created or analyzed in this study. Data sharing is not applicable to this article.
